# Association between a Variant in MicroRNA-646 and the Susceptibility to Hepatocellular Carcinoma in a Large-Scale Population

**DOI:** 10.1155/2014/312704

**Published:** 2014-08-07

**Authors:** Rui Wang, Jun Zhang, Weiru Jiang, Yanyun Ma, Wenshuai Li, Bohan Jin, Heping Hu, Jiucun Wang, Yi Liu, Jie Liu

**Affiliations:** ^1^Department of Digestive Diseases, Huashan Hospital, Fudan University, 12 Middle Wulumuqi Road, Shanghai 200040, China; ^2^Department of Immunology of Shanghai Medical School and Institutes of Biomedical Sciences, Fudan University, Shanghai 200032, China; ^3^Ministry of Education Key Laboratory of Contemporary Anthropology and State Key Laboratory of Genetic Engineering, School of Life Sciences, Fudan University, Shanghai 200433, China; ^4^Eastern Hepatobiliary Surgery Hospital, Second Military Medical University, Shanghai 200438, China

## Abstract

*Background*. Single-nucleotide polymorphisms in microRNAs play important roles in oncogenesis and cancer development. *Objective*. We aim to explore whether miR-646 rs6513497 is associated with the risk of hepatocellular carcinoma. *Methods*. Total 997 HCC patients and 993 cancer-free controls were enrolled in this study. Genotyping was performed using MassARRAY method. *Results*. Compared with the T allele of rs6513497, the G allele was associated with a significantly decreased risk of HCC (OR = 0.788, 95% CI = 0.631–0.985, *P* = 0.037); moreover, a more protective effect of the G allele was shown in males (OR = 0.695, 95% CI = 0.539–0.897, *P* = 0.005 in HCC and OR = 0.739, 95% CI = 0.562–0.972, *P* = 0.030 in HBV-related HCC), basically in a dominant manner (HCC: OR = 0.681, 95% CI = 0.162–0.896, *P* = 0.006; HBV-related HCC: OR = 0.715, 95% CI = 0.532–0.962, *P* = 0.027). *Conclusions*. Our findings support the view that the miR-646 SNP rs6513497 may contribute to the susceptibility of HCC.

## 1. Introduction

Hepatocellular carcinoma (HCC) is one of the most prevalent cancers and is the third leading cause of cancer-related death due to its extremely poor prognosis [1]. Both genetic and environmental factors can contribute to the occurrence of HCC. The chronic infection of the Hepatitis B or C virus is the main risk factor for HCC, while alcohol abuse, smoking, or exposure to toxic substances is also found to have the potential to increase the risk of HCC [2].

MicroRNA (miRNA) is a class of single-stranded, 19–25 nucleotides in length, noncoding RNA molecules in eukaryotes. Binding to the 3′-untranslated region (3′-UTR) of target mRNAs, miRNA regulates the translation and degradation of its target mRNAs and thus influences the gene expression [3, 4]. More and more evidence has shown that miRNAs are strongly related to oncogenesis [5, 6]. It has been shown that miRNAs play critical roles in tumor suppression or carcinogenesis by regulating gene expression at posttranscriptional levels [7]. A large number of tumor-derived microRNAs exist in human serum/plasma in a stable, reproducible, and consistent form, which thus can serve as potential biomarkers for blood-based detection of human cancers [8, 9]. Therefore, as a regulatory molecule, miRNA offers us a new diagnostic approach for cancers. Indeed, a recent study screened a plasma microRNA panel and identified several miRNAs which showed high effectiveness and accuracy in HCC diagnosis [10]. Such inspiring result pushed forward the development of the early detection of HCC based on microRNA.

Single-nucleotide polymorphisms (SNPs), one form of DNA variation, are pervasive in miRNAs, including pri-miRNAs, pre-miRNAs, and mature miRNAs. They could influence various biological processes by changing the secondary structure of pre-miRNAs, interfering the maturation and/or target selection of miRNAs [11, 12], and may thus play roles in the development and progression of some cancers.

Previous studies have shown that miR-646 is implicated in human cancers [13, 14]. Moreover, the SNPs in miR-646, rs112880289, rs6513496, and rs6513497, have been reported to be associated with the susceptibility to colorectal cancer [15]. Thus, it is worth questioning whether these SNPs are associated with HCC as well. However, rs112880289 is not valid in Han Chinese people while rs6513496 seems to have no effect on the structural stability of miR-646 precursor (ΔΔ*G* < 0 KJ/mol calculated by miRNASNP database [16]). Thus, the aim of this study is to investigate the clinical significance of the miR-646 SNP rs6513497 in human HCC, by analyzing a case-control Chinese population of 997 HCC patients and 993 cancer-free controls.

## 2. Materials and Methods

### 2.1. Study Population

Subjects were recruited from a case-control Chinese population as described previously [17]. In brief, patients from Huashan Hospital Affiliated to Fudan University and Eastern Hepatobiliary Surgery Hospital were diagnosed with HCC by a pathological examination or a-fetoprotein elevation (>20 ug/L) combined with imaging examination (magnetic resonance imaging, MRI and/or computerized tomography, CT). Cancer-free controls from the Taizhou Longitudinal Study had no self-reported history of cancer. All subjects including both cases and controls were unrelated Chinese Han individuals residing in East China (Shanghai, Zhejiang, Jiangsu, and Jiangxi province). All patient materials including peripheral blood samples were obtained with informed consent, and the whole procedure was approved by local ethic review committee.

### 2.2. Genotyping

Genomic DNA was isolated from the peripheral blood of the participants using AxyPrep Blood Genomic DNA Miniprep Kit (Axygen Biosciences, Union City, USA). Genotyping was done using Sequenom MassARRAY technique. The amplification primers were 5′-ACGTTGGATGCACACCTGCTTTTCACCTGT-3′ and 5′-ACGTTGGATGGTAAAGATAGGCCACTGAGC-3′, while the extension primer was 5′-CCCCCAGGAAGCAGCTGCCTC-3′.

### 2.3. Statistical Analysis

Statistical analyses were performed using SPSS (version 13.0) and Excel. Observed and expected genotype frequencies were evaluated for Hardy-Weinberg equilibrium (HWE) using Pearson's chi-square test. Odds ratio (OR) adjusted for age, gender, smoking status, and drinking status, along with 95% confidence interval (CI), was determined by logistic regression. Pearson's chi-square test was also used to assess qualitative data among different groups, while Student's *t*-test and nonparametric test were applied to compare quantitative variables. A two-sided *P* value less than 0.05 was considered statistically significant.

## 3. Results

### 3.1. Sample Overview

997 HCC patients (including 771 HBV-related HCC patients) and 993 controls were enrolled in this study. The demographic characteristics were summarized in Table 1. Briefly, it was shown that age, gender, and smoking status were significantly different between the cases and the controls (controls older than cases), while drinking status was no different between two groups.

### 3.2. Association between the miR-646 Variant and the Susceptibility to Hepatocellular Carcinoma

The genotype distributions of rs6513497 in controls, HCC patients, and HBV-related HCC patients are shown in Table 2. In the logistic regression analysis between HCC and the control subjects, it was shown that in comparison with the T allele of rs6513497, the G allele were associated with a significantly decreased risk of HCC (OR = 0.788, 95% CI = 0.631–0.985, *P* = 0.037).

In males (Table 3), some much more significant associations were found. Firstly, in comparison with the T allele of rs6513497, the G allele were associated with a greater decreased risk of HCC (adjusted OR = 0.695, 95% CI = 0.539–0.897, *P* = 0.005) and HBV-related HCC (adjusted OR = 0.739, 95% CI = 0.562–0.972, *P* = 0.030). Secondly, compared with the wild type TT of rs6513497, those with the GT genotype also have a decreased risk for HCC (adjusted OR of 0.692, 95% CI = 0.523–0.915, *P* = 0.010) and HBV-related HCC (adjusted OR of 0.719, 95% CI = 0.531–0.973, *P* = 0.033). Thirdly, we found that dominant model was suitable for the association between rs6513497 and the risk of HCC (OR = 0.681, 95% CI = 0.162–0.896, *P* = 0.006) or HBV-related HCC (OR = 0.715, 95% CI = 0.532–0.962, *P* = 0.027). Nevertheless, no association was founded in female subjects (Table 4).

### 3.3. Association between rs6513497 and Demographic Characteristic in HCC Patients

The association between rs6513497 and HCC or HBV infection was also evaluated by stratifying on other clinical indexes including total bilirubin, HBV-DNA, ALT, AST, and the number, size, and grade of tumor foci. However, no significant heterogeneity was detected between the subgroups, suggesting the independent genetic effect of rs6513497 (Table 5).

## 4. Discussion

Traditional diagnostic methods of HCC have suboptimal efficacy, sensitivity, and specificity [18, 19]. The demand of outstanding biomarkers and innovational methods with high validity and efficacy is extremely urgent [20]. The growing evidence has shown that microRNA, as a new tumor biomarker, has considerable potential to improve the accuracy of early diagnosis of HCC [21, 22]. For example, the plasma miRNA panel had high predictive accuracy for early-staged HCC [10], suggesting that miRNA could be served as a valid and noninvasive method for prediction and diagnosis of HCC. Besides, it has been well acknowledged that microRNA SNPs have relevance to the susceptibility to cancers [23, 24]. SNPs in pre-miRNAs or mature miRNAs, especially in the seed region, may interfere with miRNA processing and maturation and affect the specificity of gene silencing [25–27]. Aberrant expression of mature miRNAs or the alteration of their targets may contribute to the deregulation of relevant genes and finally lead to different cancer susceptibility, as well as different prognosis and treatment reaction [23]. Therefore, the identification of significant functional SNP sites may open a new avenue for HCC early diagnosis/predication.

MiR-646 belongs to miR-15/107 gene group which serves key functions in humans such as cell division, metabolism, stress response, and angiogenesis. Moreover, there is a seed sequence “AGCAGC” near the 5′ end of the mature miR-646 [28]. Particularly, the miR-646 SNP rs6513497, which is in the seed sequence, has been identified to be involved in the susceptibility to colorectal cancer [15, 28] and potentially to be used as a noninvasive biomarker for cervical squamous cell carcinoma [13]. The secondary structure prediction by miRNASNP database [16] showed that the structure of miR-646 with the T allele of rs6513497 has a Δ*G* of −34.2 KJ/mol, less than that with the G allele (Δ*G* = − 33.4 KJ/mol), indicating that G allele may decrease the stability of miR-646 and then result in the dysregulation of mature miR-646.

Thus, in this study, we investigated the association between rs6513497 and the susceptibility to HCC and HBV-related HCC in a large-scale population. Our findings support the view that rs6513497 was associated with HCC and HBV-related HCC. Furthermore, the protective effect of the variant genotypes of rs6513497 (GT genotype and the G allele) was more evident in male subjects, which decreases their HCC susceptibility. To the best of our knowledge, this is the first study to discover the association of miR-646 SNP with HCC susceptibility in clinical samples, suggesting that rs6513497 could be a useful marker to predict the risk of this disease. Further studies are required to explore the molecular function of this SNP. In addition, previous studies found that the SNPs of pri-miR-218 [29], miR-499 [30, 31], and miR-196a2 [32] were also associated with the risk of HCC. Their possible corporative effects and the underlying mechanisms are also worth being discovered.

Using TargetScan [33] and miRanda [34] database, we found that the potential predicated target genes of miR-646 consist of methylthioadenosine phosphorylase (MTAP), WEE1 G2 checkpoint kinase (WEE1), and cyclin D2 (CCND2), which are involved in the development of liver cancer. rs6513497 may influence the target selection of miR-646 and then alter HCC predisposition in the population with this SNP. Although there is no report or predictive record showing that targets have been gained or lost by rs6513497 so far, such speculation is still worth being explored.

In conclusion, the results of this study suggest that the miR-646 SNP rs6513497 is associated with HCC, and the G allele may be a genetic protective factor which decreases the susceptibility of HCC, especially in male subjects. To better understand the relationship between rs6513497 and cancer risk, more functional studies of miR-646 and this SNP are proposed.

## Figures and Tables

**Table 1:** General characteristics in hepatocellular carcinoma patients and controls.

	Cases (*n* = 997)	Controls (*n* = 993)	*P* value
	Number (%) or mean ± SD	Number (%) or mean ± SD	
Age (years)	54.70 ± 11.27	59.57 ± 11.65	<0.001∗
Gender			
Male	819 (82.1)	721 (72.6)	<0.001∗
Female	178 (17.9)	272 (27.4)	
Smoking status			
Never	665 (67.6)	527 (53.1)	<0.001∗
Ever	319 (32.4)	466 (46.9)	
Drinking status			
Never	734 (74.4)	732 (73.7)	0.712
Ever	252 (25.6)	261 (26.3)	
HBsAg (*n* = 951)			
Negative	180 (18.9)		
Positive	771 (81.1)		
Tumor size (*n* = 540)			
<5 cm	218 (40.4)		
≥5 cm	322 (59.6)		
Tumor number (*n* = 539)			
Single	475 (88.1)		
Multiple	64 (11.9)		
Tumor grade (*n* = 385)			
I-II	84 (21.8)		
III-IV	301 (78.2)		
Serum level of tumor markers			
ALT (U/L, in 989 subjects)	58.29 ± 86.04		
AST (U/L, in 985 subjects)	62.24 ± 81.06		
AFP			
<20 ug/L	363 (37.2)		
≥20 ug/L	612 (62.8)		
(ug/L, in 404 subjects)	126.87 ± 289.61 (0.6–1210)		
HBV-DNA (IU/mL, in 450 subjects)	1.742*E*06 ± 5.430*E*06 (1000 − 6.9*E*07)		

*P value less than 0.05.

**Table 2:** Association between genotypes/alleles of miR-646 rs6513497 and the risk of HCC.

Genotypes	Controls	HCC patients			HCC patients with HBV		
	Number (%)	Number (%)	OR (95% CI)^a^	*P* value^a^	Number (%)	OR (95% CI)^a^	*P* value^a^
miR-646 rs6513497	*n* = 993	*n* = 997			*n* = 771		
TT	795 (80.1)	825 (82.7)	1.000		639 (82.9)	1.000	
GT	186 (18.7)	166 (16.6)	0.812 (0.635–1.037)	0.095	126 (16.3)	0.814 (0.621–1.067)	0.137
GG	12 (12.2)	6 (0.6)	0.468 (0.167–1.308)	0.147	6 (0.8)	0.631 (0.222–1.793)	0.387
Dominant model (TT versus GT + GG)			0.791 (0.622–1.005)	0.055		0.803 (0.616–1.046)	0.104
Recessive model (TT + GT versus GG)			0.485 (0.174–1.355)	0.168		0.654 (0.230–1.856)	0.425
T	1776 (0.9)	1816 (0.9)	1.000		1402 (91.0)	1.000	
G	210 (0.1)	178 (0.1)	0.788 (0.631–0.985)	0.037∗	138 (9.0)	0.814 (0.637–1.039)	0.098

^a^ORs and *P* values were all obtained after adjusting for age, gender, smoking status, and wine status.

*P value less than 0.05.

**Table 3:** Comparison of genotype/allele frequencies of miR-646 rs6513497 in male subjects.

Genotypes	Controls	HCC patients			HCC patients with HBV		
	Number (%)	Number (%)	OR (95% CI)^a^	*P*-value^a^	Number (%)	OR (95% CI)^a^	*P* value^a^
miR-646 rs6513497	*n* = 721	*n* = 819			*n* = 643		
TT	570 (79.1)	685 (83.6)	1.000		536 (83.4)	1.000	
GT	143 (19.8)	129 (15.8)	0.692 (0.523–0.915)	0.010∗	102 (15.9)	0.719 (0.531–0.973)	0.033∗
GG	8 (1.1)	5 (0.6)	0.492 (0.151–1.596)	0.237	5 (0.7)	0.650 (0.198–2.134)	0.478
Dominant model (TT versus GT + GG)			0.681 (0.517–0.896)	0.006∗		0.715 (0.532–0.962)	0.027∗
Recessive model (TT + GT versus GG)			0.525 (0.162–1.701)	0.023∗		0.690 (0.211–2.259)	0.540
T	1283 (89.0)	1499 (91.5)	1.000		1172 (91.3)	1.000	
G	159 (11.0)	139 (8.5)	0.695 (0.539–0.897)	0.005∗	112 (8.7)	0.739 (0.562–0.972)	0.030∗

^a^ORs and *P* values were obtained after the adjustment of age, gender, smoking status, and wine status.

*P value less than 0.05.

**Table 4:** Comparison of genotype/allele frequencies of miR-646 rs6513497 in female subjects.

Genotypes	Controls	HCC patients			HCC patients with HBV		
	Number (%)	Number (%)	OR (95% CI)^a^	*P* value^a^	Number (%)	OR (95% CI)^a^	*P* value^a^
miR-646 rs6513497	*n* = 272	*n* = 178			*n* = 128		
TT	225 (82.7)	140 (78.7)	1.000		103 (80.5)	1.000	
GT	43 (15.8)	37 (20.8)	1.355 (0.817–2.246)	0.239	24 (18.8)	1.318 (0.727–2.389)	0.363
GG	4 (1.5)	1 (0.5)	0.409 (0.0443–0.799)	0.431	1 (0.7)	0.597 (0.061–5.833)	0.657
Dominant model (TT versus GT + GG)			1.274 (0.777–2.088)	0.377		1.254 (0.702–2.240)	0.444
Recessive model (TT + GT versus GG)			0.386 (0.042–3.579)	0.402		0.568 (0.058–5.534)	0.626
T	493 (90.6)	317 (89.0)	1.000		230 (89.8)	1.000	
G	51 (9.4)	39 (11.0)	1.177 (0.746–1.855)	0.484	26 (10.2)	1.183 (0.693–2.018)	0.538

^a^ORs and *P* values were obtained after adjusting for age, gender, smoking status, and wine status.

**Table 5:** Clinicopathologic characteristics and genotype/allele frequencies of miR-646 rs6513497 in HCC patients.

Indexes	Genotype			*P* value	Allele		*P* value
Tumor size							
miR-646 rs6513497	TT	GT	GG	0.713	T	G	0.617
<5 cm	180 (84.1)	35 (16.9)	0 (0)		395 (31.9)	35 (68.1)	
≥5 cm	261 (82.6)	53 (16.8)	2 (0.6)		575 (34.3)	57 (65.7)	
Tumor focus number							
miR-646 rs6513497	TT	GT	GG	0.195	T	G	0.343
Single	396 (83.4)	78 (16.4)	1 (0.2)		870 (91.6)	80 (8.4)	
Multiple	51 (79.7)	12 (18.8)	1 (1.5)		114 (89.1)	14 (10.9)	
Tumor grade							
miR-646 rs6513497	TT	GT	GG	0.897	T	G	0.771
I-II	71 (84.5)	13 (15.5)	0 (0)		155 (92.3)	13 (7.7)	
III-IV	250 (83.1)	50 (16.6)	1 (0.3)		550 (91.4)	52 (8.6)	
AFP							
miR-646 rs6513497	TT	GT	GG	0.548	T	G	0.371
<20 ug/L	294 (81.0)	67 (18.5)	2 (0.6)		655 (90.2)	71 (9.8)	
≥20 ug/L	511 (83.5)	97 (15.8)	4 (0.7)		1119 (91.4)	105 (8.6)	
Total bilirubin							
miR-646 rs6513497	TT	GT	GG	0.184			
	18.79 ± 0.94	20.12 ± 3.18	21.90 ± 2.86				
Direct bilirubin							
miR-646 rs6513497	TT	GT	GG	0.347			
	8.78 ± 0.70	9.91 ± 2.47	8.43 ± 1.32				
Indirect bilirubin							
miR-646 rs6513497	TT	GT	GG	0.125			
	9.81 ± 0.24	10.22 ± 0.77	13.50 ± 1.78				
ALT							
miR-646 rs6513497	TT	GT	GG	0.643			
	57.14 ± 2.79	59.92 ± 7.51	51.38 ± 8.13				
AST							
miR-646 rs6513497	TT	GT	GG	0.730			
	62.17 ± 2.78	63.58 ± 7.04	41.50 ± 3.90				
HBV-DNA							
miR-646 rs6513497	TT	GT	GG	0.526			
	1.73*E*06 ± 2.85*E*05	1.73*E*06 ± 6.15*E*05	2.93*E*06 ± 1.68*E*06				

## References

[1] Jemal A, Bray F, Center MM, Ferlay J, Ward E, Forman D (2011). Global cancer statistics. *CA Cancer Journal for Clinicians*.

[2] Gramantieri L, Fornari F, Callegari E (2008). MicroRNA involvement in hepatocellular carcinoma. *Journal of Cellular and Molecular Medicine*.

[3] Kertesz M, Iovino N, Unnerstall U, Gaul U, Segal E (2007). The role of site accessibility in microRNA target recognition. *Nature Genetics*.

[4] Bartels CL, Tsongalis GJ (2009). MicroRNAs: novel biomarkers for human cancer. *Clinical Chemistry*.

[5] Miska EA (2005). How microRNAs control cell division, differentiation and death. *Current Opinion in Genetics and Development*.

[6] Watanabe Y, Kanai A (2011). Systems biology reveals microRNA-mediated gene regulation. *Frontiers in Genetics*.

[7] Jiang YW, Chen LA (2012). MicroRNAs as tumor inhibitors, oncogenes, biomarkers for drug efficacy and outcome predictors in lung cancer (review). *Molecular Medicine Reports*.

[8] Mitchell PS, Parkin RK, Kroh EM (2008). Circulating microRNAs as stable blood-based markers for cancer detection. *Proceedings of the National Academy of Sciences of the United States of America*.

[9] Chen X, Ba Y, Ma L (2008). Characterization of microRNAs in serum: a novel class of biomarkers for diagnosis of cancer and other diseases. *Cell Research*.

[10] Zhou J, Yu L, Gao X (2011). Plasma microRNA panel to diagnose hepatitis B virus-related hepatocellular carcinoma. *Journal of Clinical Oncology*.

[11] Duan R, Pak C, Jin P (2007). Single nucleotide polymorphism associated with mature miR-125a alters the processing of pri-miRNA. *Human Molecular Genetics*.

[12] Xu T, Zhu Y, Wei Q (2008). A functional polymorphism in the miR-146a gene is associated with the risk for hepatocellular carcinoma. *Carcinogenesis*.

[13] Wang WT, Zhao YN, Yan JX (2014). Differentially expressed microRNAs in the serum of cervical squamous cell carcinoma patients before and after surgery. *Journal of Hematology & Oncology*.

[14] Cummins JM, He Y, Leary RJ (2006). The colorectal microRNAome. *Proceedings of the National Academy of Sciences of the United States of America*.

[15] Venkatachalam R, Verwiel ETP, Kamping EJ (2011). Identification of candidate predisposing copy number variants in familial and early-onset colorectal cancer patients. *International Journal of Cancer*.

[16] Gong J, Tong Y, Zhang H (2012). Genome-wide identification of SNPs in MicroRNA genes and the SNP effects on MicroRNA target binding and biogenesis. *Human Mutation*.

[17] Jun Zhang RW, Ma Y, Chen L (2013). Association between the single nucleotide polymorphisms in miRNA196a-2 and miRNA146a and susceptibility to hepatocellular carcinoma in a Chinese population. *Asian Pacific Journal of Cancer Prevention*.

[18] Zhang LS, Liang WB, Gao LB (2012). Association between pri-mir-218 polymorphism and risk of hepatocellular carcinoma in a han Chinese Population. *DNA and Cell Biology*.

[19] Zhang Y, Wu H, Shen J (2007). Predicting hepatocellular carcinoma by detection of aberrant promoter methylation in serum DNA. *Clinical Cancer Research*.

[20] Chen CJ, Lee MH (2011). Early diagnosis of hepatocellular carcinoma by multiple microRNAs: validity, efficacy, and cost-effectiveness. *Journal of Clinical Oncology*.

[21] Iorio MV, Croce CM (2012). MicroRNA dysregulation in cancer: diagnostics, monitoring and therapeutics. A comprehensive review. *EMBO Molecular Medicine*.

[22] Corsini LR, Bronte G, Terrasi M (2012). The role of microRNAs in cancer: diagnostic and prognostic biomarkers and targets of therapies. *Expert Opinion on Therapeutic Targets*.

[23] Slaby O, Bienertova-Vasku J, Svoboda M, Vyzula R (2012). Genetic polymorphisms and microRNAs: new direction in molecular epidemiology of solid cancer. *Journal of Cellular and Molecular Medicine*.

[24] Lv M, Dong W, Li L (2013). Association between genetic variants in pre-miRNA and colorectal cancer risk in a Chinese population. *Journal of Cancer Research and Clinical Oncology*.

[25] Duan S, Mi S, Zhang W, Dolan ME (2009). Comprehensive analysis of the impact of SNPs and CNVs on human microRNAs and their regulatory genes. *RNA Biology*.

[26] Nicoloso MS, Sun H, Spizzo R (2010). Single-nucleotide polymorphisms inside microRNA target sites influence tumor susceptibility. *Cancer Research*.

[27] Ryan BM, Robles AI, Harris CC (2010). Genetic variation in microRNA networks: the implications for cancer research. *Nature Reviews Cancer*.

[28] Finnerty JR, Wang W, Hébert SS, Wilfred BR, Mao G, Nelson PT (2010). The miR-15/107 group of MicroRNA genes: evolutionary biology, cellular functions, and roles in human diseases. *Journal of Molecular Biology*.

[29] Han Y, Pu R, Han X (2014). Association of a potential functional pre-miR-218 polymorphism and its interaction with hepatitis B virus mutations with hepatocellular carcinoma risk. *Liver International*.

[30] Ma Y, Wang R, Zhang J (2014). Identification of miR-423 and miR-499 polymorphisms on affecting the risk of hepatocellular carcinoma in a large-scale population. *Genetic Testing and Molecular Biomarkers*.

[31] Zou H, Zhao Y (2013). Positive association between miR-499A > G and hepatocellular carcinoma risk in a chinese population. *Asian Pacific Journal of Cancer Prevention*.

[32] Zhang J, Wang R, Ma YY (2013). Association between single nucleotide polymorphisms in miRNA196a-2 and miRNA146a and susceptibility to hepatocellular carcinoma in a Chinese population. *Asian Pacific Journal of Cancer Prevention*.

[33] Lewis BP, Burge CB, Bartel DP (2005). Conserved seed pairing, often flanked by adenosines, indicates that thousands of human genes are microRNA targets. *Cell*.

[34] John B, Enright AJ, Aravin A, Tuschl T, Sander C, Marks DS (2005). Correction: human microRNA targets. *PLoS Biology*.

